# In AI We Trust: Ethics, Artificial Intelligence, and Reliability

**DOI:** 10.1007/s11948-020-00228-y

**Published:** 2020-06-10

**Authors:** Mark Ryan

**Affiliations:** The Division of Philosophy, KTH Royal Institute of Technology, Stockholm, Sweden

**Keywords:** Artificial intelligence ethics, Trustworthy AI, European commission high-level expert group, Philosophy of trust, Reliability

## Abstract

One of the main difficulties in assessing artificial intelligence (AI) is the tendency for people to anthropomorphise it. This becomes particularly problematic when we attach human moral activities to AI. For example, the European Commission’s High-level Expert Group on AI (HLEG) have adopted the position that we should establish a relationship of trust with AI and should cultivate trustworthy AI (HLEG AI Ethics guidelines for trustworthy AI, 2019, p. 35). Trust is one of the most important and defining activities in human relationships, so proposing that AI should be trusted, is a very serious claim. This paper will show that AI cannot be something that has the capacity to be trusted according to the most prevalent definitions of trust because it does not possess emotive states or can be held responsible for their actions—requirements of the affective and normative accounts of trust. While AI meets all of the requirements of the rational account of trust, it will be shown that this is not actually a type of trust at all, but is instead, a form of reliance. Ultimately, even complex machines such as AI should not be viewed as trustworthy as this undermines the value of interpersonal trust, anthropomorphises AI, and diverts responsibility from those developing and using them.

## Introduction

One of the main difficulties with analysing the ethical impact of artificial intelligence (AI) is overcoming the tendency to anthropomorphise it. The media is enthralled by images of machines that can do what we can, and often, far better. We are bombarded with novels, movies and television shows depicting sentient robots, so it is not surprising that we associate, categorise, and define these machines in human terms. While people associate human activities and abilities to machines, it becomes problematic when this anthropomorphisation is attached to human moral activities, such as trust.

Organisations, such as the European Commission’s High-level Expert Group on AI (HLEG),^1^ have adopted the position that AI is something that we can, and should, trust (HLEG 2019, p. 35). However, this requires that ‘*all* actors and processes [including the AI technology itself] that are part of the system’s socio-technical context throughout its entire life cycle [emphasis added]’ are trustworthy (HLEG 2019, p. 5). The HLEG state that while trustworthiness is not typically a property ascribed to machines, they want to ascribe it to AI. They propose that there are three main characteristics of trusting AI:

- The AI technology itself;
- Designers and organisations behind the development, deployment and use of AI; and
- The socio-technical systems involved in the AI life cycle (HLEG 2019, p. 5).^2^

While the second and third components are important, the first proposal is a far more radical claim. Trust is one of the most important and defining activities in human relationships, so proposing that AI should be trusted, is a very serious claim. This paper will reject the position taken by the HLEG, and many within the academic field, that AI technology is something that has the capacity to be trusted, and thus, undermining the fact that it can be trustworthy.

This paper begins by defining AI, specifically the difference between narrow and general AI. Section Two provides an overview of the three main accounts of trust: rational, affective, and normative. Section Three establishes a definition of trust based on five characteristics: A has confidence in B to do X; A believes in the competence of B to do X; A is vulnerable to the actions of B; there is a possibility that B will betray A’s trust; and A believes that B is motivated to do X for affective or normative reasons.

This paper will show that AI cannot be something that has the capacity to be trusted according to the most prevalent definitions of trust because it does not possess emotive states or be held responsible for its actions—requirements of the affective and normative accounts of trust. While AI meets all of the requirements of the rational account of trust, this is not a type of trust at all, but is instead, a form of reliance. Ultimately, AI should not be viewed as trustworthy because it undermines the value of interpersonal trust, anthropomorphises AI (the affective account of trust), and diverts responsibility from those developing and using AI (the normative account of trust).

### Artificial Intelligence (AI)

The definition of AI is a highly contested concept. It often refers to technologies that demonstrate levels of independent intelligence from humans. By its very definition, it is an intelligence that is differentiated from natural intelligence; it is a constructed, artificial, or machine intelligence. AI are systems that are designed by human beings that can facilitate complex tasks, and can process information in a similar way to us. It is a field of computer science that focuses on computer processes that can often function and react in human-like ways; such as image recognition (*vision*), speech recognition (*hearing*), and natural language generation (*speaking*). AI is artificial mimicry of tasks and functions that would otherwise require human intelligence (Department for Business, Energy, and Industrial Strategy 2017).

AI is used in the healthcare sector to predict the onset of diseases (Yan et al. 2019), to detect fraud in insurance (Kancevičienė 2019), to predict crime in law enforcement (Asaro 2019), in agriculture to increase crop yields (Ryan 2019b, 2020), in cities to reduce congestion (Ryan 2019a; Ryan and Gregory 2019), and in logistics to identify supply-chain management risks (Jiya 2019). Not only is AI being used for raw data processing and prediction, it is also being embodied in physical constructs, such as robots, drones, and self-driving cars. However, not all robots and drones have AI. We can also have ‘dumb’ robots, such as those used for bomb disposals, factories, and manufacturing plants. In fact, only a small percentage of robots have AI. While embodied AI is an important area of research, it often dominates the debate because it is one of the most tangible and fascinating areas for the general public.

Another important issue is the differentiation between narrow AI and general AI. Narrow AI is a system that is designed and used for specific or limited tasks (Macnish et al. 2019). It is a type of analytical intelligence that is used for defined functions and applications (Kaplan and Haenlein 2019). Whereas, general AI, or artificial general intelligence (AGI), ‘is an AI system with generalized human cognitive abilities. When presented with an unfamiliar task, a strong AI system is able to find a solution without human intervention’ (SearchEnterpriseAI 2019). General AI has similar cognitive abilities as humans and can convincingly demonstrate these capacities (UK House of Lords 2018, p. 21). The focus of this paper is narrow AI because it is the focus of the HLEG and also because general AI is still speculative.

### Theories of Trust

Trust underpins many aspects of our lives and is necessary for some of the most fundamental relationships in the human lifespan. We trust our partners to be faithful, we trust that our friends will keep our secrets, and we trust our family members to stand by us in difficult times and situations. Trust may be one of the most foundational attitudes or activities within human interaction and without it, many important social bonds would be jeopardised. Without at least a minimal amount of trust in others, we would become paranoid and isolationist because of fear of deceit and harm (O’Neill 2002, p. 12).

The placement of trust in someone often requires a belief about their trustworthiness, but the two are not synonymous: ‘Patients can misplace trust in physicians or institutions that are not deserving, or they can fail to trust those that are’ (Hall et al. 2001, p. 616). Trusting someone is placing a confidence in them to carry out a particular action. ‘Being trustworthy helps in gaining trust, but is neither necessary nor sufficient. Deceivers can attract others’ trust, so *misplaced trust* is common enough. The trustworthy can be denied others’ trust, so *misplaced mistrust* is also common enough’ (O’Neill 2002, p. 165). Trustworthy agents are those worthy of being trusted—whether or not we trust them. To be worthy of trust, though, one must be capable of being trusted. Trustworthy agents are those who have the competence to actually fulfil the trust that is placed in them. This paper will analyse whether AI is something that can be trusted, and something worthy of our trust. If it cannot, then all placement of trust in AI would be *misplaced trust*.

To begin with, some claim that we should not view trust with such strong moral connotations and that we can trust many things in our everyday activities. For example, Mark Coeckelbergh claims that while artefacts, such as AI, do not meet the specific criteria for trust, we may still trust them because ‘they may nevertheless contribute to the establishment of ‘virtual trust’ or ‘quasi trust’ in so far that they appear as quasi-others or social others’ (Coeckelbergh 2012, p. 57). It is irrelevant whether AI has the capacity to be trusted, it simply depends on whether the trustor believes it (Coeckelbergh 2009). Certainly, one *can* say that they trust artefacts, such as AI, but this type of ‘quasi trust’ is actually *misplaced trust*. This type of misplaced trust has the potential to deceive individuals about the capacities of AI and obfuscate responsibility by AI companies (which will be discussed in the affective and normative sections, respectively). I will show that this type of quasi trust relates to only the ‘rational account’ of trust.

This paper will evaluate three dominant trust paradigms to analyse if AI can be something that has the capacity to be trusted: rational, affective, and normative accounts. It will be shown that AI only meets the requirements of the rational account, which is in fact a form of reliance because of its lack of concern about the trustee’s motivation for action.

The rational account of trust states that the trustor is making a logical choice, weighing up the pros and cons, when determining whether to place their trust in the trustee. It is a rational calculation of whether the trustee is someone that will uphold the trust placed in them (Mollering 2006). Trust is simply a matter of prediction by the trustor, rather than being concerned about the trustee’s motivation (Nickel et al. 2010).

The affective account of trust states that the trustor places a confidence in, and belief in, the goodwill of the trustee. There is an ‘expectation that the one trusted will be directly and favourably moved by the thought that we are counting on her’ (Jones 1996, p. 4; see also Baier 1986). Ultimately, the defining feature of the affective account of trust is that the motivation of B to do X is based on a goodwill towards A.

Trust based on the normative account implies that the trustee’s actions will be grounded on what they *ought to do*. The expectation placed on the trustee is not only what they will do, but what they should do. ‘In other words, we have normative, rather than merely predictive, expectations of them. […] then to be trustworthy is to live up to these expectations, and a failure to do so can result in betrayal’ (McLeod 2015).

The definitions include three characteristics: A has confidence in B to do X; A believes B is competent to do X; A is vulnerable to the actions of B. After this initial characterisation, they differ. The affective and normative accounts state that if B does not do X then A may feel betrayed, whereas, this is not included in the rational account. Furthermore, all three definitions vary in their views on the motivation of B, with the rational account not requiring a motive, the affective account basing it on a goodwill towards A, and the normative account basing it on a normative commitment to their relationship with A.

In the following section, I will evaluate these three positions by analysing their defining characteristics to determine if they can be applied to AI technology. The three accounts of trust are not necessarily mutually exclusive. One may combine all three positions to determine who to trust. I will analyse all of the individual components of the three definitions for the purpose of identifying if AI has the capacity to be trusted under these three positions.

### Interpersonal Trust and AI

This paper proposes a definition of interpersonal trust (A trusts B) found within all three accounts of trust. As the three definitions of trust have largely the same characteristics, those components will be evaluated collectively and when they differ, those sections will be analysed separately (e.g. the motivation of the trustee). The characteristics of trust are:

i. A has confidence in B to do X.
ii. A believes B is competent to do X.
iii. A is vulnerable to the actions of B.
iv. If B does not do X then A may feel betrayed.
v. A thinks that B will do X, motivated by *one* of the following reasons:
  i. Their motivation does not matter (rational trust)
  ii. B’s actions are based on a goodwill towards A (affective trust)
  iii. B has a normative commitment to the relationship with A (normative trust)

Each of these distinctions is based on a sliding-scale, where there are varying degrees and contexts of trust. However, they must contain many of these components to be classified as trust (see Table 1). The degree of these characteristics vary, depending on the context and relationship at stake. For example, one may trust their partner to remain monogamous, despite previously cheating. There may be high levels of vulnerability and betrayal, but low levels of confidence in the trustee. Or one’s competence to stay monogamous is low because one is a sex addict. One may have high levels of confidence in a friend to keep a secret, but the levels of vulnerability and betrayal are low because the secret is about someone else.

**Table 1:** Components of interpersonal trust

Trust component	Rational trust	Affective trust	Normative trust
Confidence	√	√	√
Competence	√	√	√
Vulnerability	√	√	√
Betrayal		√	√
Motivation		Affective	Normative

The following sections will outline the different components within trust definitions and will propose that AI can meet the first three requirements, but fails to meet the last two. AI can only meet the requirements of the rational account (see Table 1). However, this is not a type of trust at all, but in fact, a form of reliance. However, this does not mean that we should distrust AI: ‘In between trust and distrust are found various forms of relying on and taking for granted’ (Jones 1996, p. 16). AI is something we can have a reliance on, but not something that has the capacity to be trusted.

#### A has Confidence in B to do X

One component of trust is that there is typically a *confidence* placed in the trustee to do something^3^: A trusts B to do X (Hardin 2002, p. 9). For example, I trust my partner to be faithful to me; or I trust my friend to keep my secret. The trustor places a confidence in, and thinks well of, the trustee to do the action that is entrusted to them. ‘The *expectations* the trustor has of the trustee have to be positive and favourable’ (Keymolen 2016, p. 14; see also Luhmann 1979). One must have a sense of confidence in the person doing X, because there can be no breach of trust if I am pessimistic that B will do X.

For example, if my friend states that he will climb the summit of Mount Everest with me, but I have no confidence that he will do this, then I cannot be said to trust him to do this. While he promises me that he will do it, he always makes ludicrous goals and never follows through. He may prove me wrong, but it is very unlikely. For me to trust my friend in this situation, he would have to instil a confidence in me that he will do X. I cannot trust him if I did not believe that he would do X in the first place. This component of trust evaluates the likelihood that an agent will carry out the action in question.^4^

While there may be types of AI that we are less confident in, this does not necessarily mean that we are pessimistic about AI, generally. When we get into a self-driving vehicle, we may be confident that the AI systems used in it will bring us safely to our destination; when we allow AI robots in nursing homes, we are confident that they will be beneficial for patient care; and when we use AI to identify potential customers, we are confident that the results are somewhat accurate. However, we can also imagine the converse of this to be true. People may be fearful of getting into self-driving cars, refuse AI robots in elderly care facilities, or fear incorporating AI into one’s business model.

The Pew Research Centre conducted a survey in 2018^5^ with 979 technology experts, developers, innovators, business leaders, and activists: 63% believed that people would be better off, while 37% stated that people would not be better off, from using AI (Anderson and Rainie 2018). In a recent survey of 1000 US citizens, 50% stated that they were fearful about AI (Blumberg Capital 2019). While these results are startling, it does not mean that it is impossible to increase confidence in AI. Society would have previously lacked a confidence in telephones, trains, print, and steam ships, when they were first invented; but now they are used throughout the world (Higgitt 2013). Even if there is a low confidence in AI now, this does not mean that it cannot be changed in the future.

#### A Believes B is Competent to do X

B needs to be *competent* to do X. B does not have to be competent to do everything, but simply the act that they are entrusted with. The trust placed in them to do X has to be within their capacity. Having the competence and ability to do X is one characteristic that distinguishes trust from [mere] *hopefulness*. Competence can mean physical, emotional or moral competence to fulfil the entrusted activity. For example, the trust that I place in my partner to be faithful, or the trust that I place in my friend to keep my secret, [I believe] are within their capacities. But what about my Everest-climbing friend? What if I am bowled over by his enthusiasm and develop a confidence that he will climb the peak? However, I am also aware of the physical competence required to achieve this specialised and difficult task. While I believe in his moral competence to keep his promise, it would be difficult to trust him in this circumstance because of his striking lack of competences to fulfil this activity.

Trust may require one or several competences to fulfil an activity. In the Everest example, while my friend possessed the moral competence, he strongly lacks the physical competence, required to fulfil this activity. For AI to be trusted, it needs to have the competence to fulfil the action entrusted to it. This would mean that AI in self-driving vehicles should have the capacity to bring individuals safely to their destination, AI used in the insurance industry should accurately detect fraud claims, and AI used in healthcare should accurately predict the onset of tumours. One of the main reasons for the promotion of AI is that it can, and will, be able to do tasks much faster and more effectively than humans. While some AI applications are insufficient now (e.g. AI chess-playing in the early 1990s), there is no reason that they will not be in the future (e.g. AI chess-playing now, see Gibbs 2017). This is fundamentally an issue of technological robustness of AI, rather than a deeply rooted philosophical problem—with the exception of moral competence, which will be discussed in Section V.

#### A is Vulnerable to the Actions of B

While I have optimism in the trustee, and I believe that they are competent, there is still a *risk* that they will not, for whatever reason, fulfil X. There is the possibility that the trustee will breach the trust placed in them. Trust incorporates optimistic feelings about a particular future occurring, where the trustee behaves as we had hoped. The risk involved will differ from situation-to-situation, and often, the degree of risk to the trustor may determine the level of trust placed in the trustee; for example, if I trust you with my life (Luhmann 1979, p. 43). Trust allows us to deal with uncertainty and risk. However, we do not ignore uncertainty, but rather, we overcome some of the fears surrounding it: ‘There is never enough information to give assurance and let complexity dissolve. Trust reduces complexity; it does not take it away’ (Keymolen 2016, p. 45).

The trustor is vulnerable because they are placing their faith in the trustee. While I trust my friend to keep my secret, my friend to climb Mount Everest with me, and my partner to remain monogamous, there is still a risk that they may not.^6^ There is a risk that they will breach my trust, so I am vulnerable to their actions. ‘To trust someone means to be vulnerable and dependent on the action of a trustee who in his turn can take advantage of this situation of vulnerability and betray the trustor’ (Keymolen 2016, p. 36). One is not trying to avoid or overcome one’s vulnerability, but instead, there is a positive acceptance of it. Trust in others is used as a way to plan for the future as if it were certain, despite being aware that it is not (Luhmann 1979, p. 10). However, it is the ‘as if’ that truly defines trust because it becomes ‘redundant when action or outcomes are guaranteed’ (O’Neill 2002, p. 13). Trust is the positive expectation that a certain reality will materialise—namely, that the trustee will not breach our trust (Keymolen 2016, p. 15). Essentially, ‘trust is inseparable from *vulnerability*, in that there is no need for trust in the absence of vulnerability’ (Hall et al. 2001, p. 615).

AI is being used in most fields and industries and its widespread adoption is only set to increase. We will be physically vulnerable to autonomous vehicles driving us to our location, emotionally vulnerable to robots in healthcare settings, and financially vulnerable to AI in the insurance and banking sectors. If anything, our vulnerability to AI is one of the driving factors behind the need for ensuring that it is developed, deployed and used in an ethical way. We are vulnerable to the effects of AI because of the tasks that are delegated to it, but individuals who do not choose to delegate tasks to AI will also be vulnerable.^7^

#### There is a Possibility that B will Betray A’s Trust

There is a risk that our trust will be breached, resulting in a cost to the trustor—a betrayal of their trust (Tuomela and Hofmann 2003, p. 167). Betrayal closely relates to the confidence placed in, and competence of, the trustee. For example, when my friend gets drunk, he often loses control of what he is saying (i.e. *competency*). If he tells my secret while he is drunk, I will feel disappointed and betrayed because I thought that I could trust him. However, I might feel greater levels of betrayal if another friend did this, because they do not suffer from the same problem when drinking, all else being equal. I may feel diminished levels of betrayal, but greater levels of disappointment, when I find out that my partner was unfaithful to me for a second time. While I trusted her when she said that it would never happen again, my *confidence* in her was diminished.^8^ However, how can we distinguish between betrayal and disappointment? The following examples may help illustrate this comparison with AI:

- My computer usually works fine for me, but yesterday it would not turn on.
- I use the elevator to get to work, but today it is out of order.
- I often leave my dog left alone in the house. However, while I was at work yesterday, he tore up my couch.

Feelings of betrayal are not typically associated to dogs, elevators or computers. While we may have believed in the competences and felt optimistically towards them (similarly to AI), this is not based on our trust in them. At most, we can only feel justly *disappointed* by them, or more likely, a disappointment in the situation that has occurred. This is because disappointment is the appropriate response when someone simply relied on someone or something to do X, rather than trusting them to do it. We feel disappointed by people or things that we rely on, while we feel betrayed by those we trust (Holton 1994). Reliance is a property of relations that something is supposed to carry out, while reliability is the capacity of that thing to achieve that ends (Fossa 2019, p. 70). However, reliance and trust are often conflated, so it is important to clearly identify the differences.

Potter (2002) provides an example to demonstrate the difference between trustworthiness and reliability: There is a sexist employer who treats his female staff well because he fears legal sanctions if he does not. Because he has not done anything inappropriate to his current female employees, they may consider him reliable, but not trustworthy. ‘The female employees might know that their employer treats them well only because he fears social sanctioning. In that case, he could not betray them [because they did not place any trust in him to begin with], although he could disappoint them’ (McLeod 2015). However, the rational account of trust would state that the female employees can trust the sexist boss because this type of trust only focuses on the trustee’s past behaviour to predict whether they should be trusted.

Tavani (2015) gives another example to demonstrate the difference between feelings of betrayal and disappointment in AI. Tavani has an autonomous vehicle in the future, where they have installed a chauffeur-like ‘Johnny-Bot’ to put his mind at ease by having an anthropomorphic AI driver. He gives instructions to the Johnny Bot to drive through a red light (because his wife, Joanne, needs to be rushed to the hospital), which the AI refuses to do because his job is to protect the passengers in the vehicle. The robot also resists Tavani’s attempts to physically take control of the car (p. 85). Johnny Bot has an explicit decision-making capacity and Tavani claims that he felt let down by ‘him’ (Johnny Bot).

This is very clearly a demonstration of what O’Neill (2002) referred to as *misplaced trust*: ‘it would seem that I may have placed a degree or level of trust in Johnny that was not warranted’ (Tavani 2015, p. 85). However, Tavani admits that it would be *unfair* to say that Johnny Bot betrayed his trust, but instead, it only disappointed him because of its limited autonomy and ‘he could not freely have done other than what he did in that particular situation, given the specific software programming code built into him’ (p. 86). This is because only full moral agents are capable of betraying one’s trust.

The levels of trust one can place in another human being are full and complete because of their full moral agency status. One can only feel disappointed by AI, because this ‘refers to functional expectations that are not met and, as such, is the *appropriate reaction* to reliability issues’ (Fossa 2019, p. 75). As I have already demonstrated in this section, we feel disappointed by those we rely on (e.g. AI), but feel betrayed by those we trust (e.g. fellow human beings). The exclusion of betrayal is incompatible with the normative and affective accounts of trust, but not necessarily the rational account of trust. However, the exclusion of betrayal from definitions of trust lead to dubious and incoherent conclusions, as demonstrated in this section.

#### A thinks that B will do X, motivated by Y

What distinguishes the affective and normative accounts from the rational account is that they state that betrayal can be distinguished from mere disappointment by the allocation of intent of the trustee. For example, I relied on the computer to work correctly, my dog to behave appropriately, or the sexist employer to act fairly because of sanctions, rather than being motivated by a goodwill towards the trustor (Baier 1986; and Jones 1996), or their normative commitment to them (O’Neill 2002; Simpson 2012; and Walker 2006). I may have relied on my computer to function correctly, or for my dog to not tear up my furniture, or the sexist employer to act appropriately, but we cannot be said to have trusted them. This is because of the trustee’s motivation for action, which is lacking in the rational account.

##### Trust Based on the Rational Account

The rational account of trust can only be classified as relying on B to perform X, which is made out of a rational prediction that they will do it. It is a ‘reliance on another person’s qualities or features of the situation, disregarding the trustor’s social right embedded in their relationship of mutual respect to have the trustee’s responsiveness to general social rights involved in that type of relationship’ (Tuomela and Hofmann 2003, p. 164). However, reliability is only one factor used to determine whether to trust an agent: ‘In judging that someone is reliable we look to their past performance; in placing trust in them we commit ourselves to relying on their future performance’ (O’Neill 2002, p. 14).

If one only focuses on reliability, then in certain situations we may not be able to trust; for example, establishing amnesties, peace treaties, and agreements with those who have broken our trust in the past: ‘We can see that knowledge of others’ reliability is not necessary for trust by the fact that we can place trust in someone with an indifferent record for reliability, or continue to place trust in others in the face of some past unreliability’ (O’Neill 2002, p. 14). Trust is separate from risk analysis that is solely based on predictions based on past behaviour (i.e. the rational account of trust). While reliability and past experience may be used to develop, confer, or reject trust placed in the trustee, it is not the sole or defining characteristic of trust. Though we may trust people that we rely on, it is not presupposed that we do.

While reliability is based on past performance, it is not the *only* thing considered, *or at all*, when trusting someone (O’Neill 2002). O’Neill claims that we would ‘expect competent persons to converge in judgements of reliability if they have access to the same evidence; we do not expect the same convergences in placing of trust’ (O’Neill 2002, p. 15). Therefore, reliability is solely grounded on predictions based on past actions; whereas, ‘[p]lacing trust is not dictated by what has happened: it is given, built and conferred, refused and withdrawn, in ways that often go beyond or fall short of that evidence’ (O’Neill 2002, p. 15). The rational version of trust is reliant on specific features of a situation, rather than the relationship between the trustor and trustee. Therefore, this type of *trust* should not be called trust at all, as it is a form of reliance (Tuomela and Hofmann 2003, p. 168). Therefore, in this account, we can only be said to have a reliance on AI, rather than a trust in it.

##### Trust Based on an Affective Account

We have different expectations of trust from different people in our lives, depending on our relationship, proximity, level of obligation, context and types of trust. Ultimately, ‘trust is composed of two elements: an affective attitude of confidence about the goodwill and competence of another as it extends to the domain of our interaction and, further, an expectation that the one trusted will be directly and favorably moved by the thought that you are counting on them’ (Jones 1996, p. 11).

Jones (1996) states that trustees are those that have our interests at heart when doing X. Their actions are fundamentally based and guided by a sense of goodwill towards us. This does not mean that a goodwill towards us has to be the *sole* reason for action, but it should take a predominant place within their actions. For example, my friend should keep my secret because he cares about me or respects my personal privacy, rather than because it will be beneficial for him to do so or he will get in trouble if he does not. My partner should remain faithful to me because of the hurt it would cause me, rather than because she is worried about finding new accommodation if we break up. The trustee should do X out of a sense of goodwill towards the trustor, rather than for personal benefit, coercion, or out of habit. Ultimately, there are three components within the affective account of trust:

- the trustee is favourably moved by the trust placed in them;
- the trustee has the trustor’s interests at heart; and
- the trustee is motivated out of a sense of goodwill to the trustor.

The trustee needs to be aware that the trustor is counting on them, and be moved by this, to act in a way that upholds the trust placed in them. This is not proposing that the trustee is a blind servant to the needs of the trustor, rather they have to be motivated by the trustor’s interests when carrying out the entrusted activity. One needs to be freely motivated to carry out the entrusted action and be moved by the fact the trustor is counting on them. The trustee must have an emotive state that makes them favourably moved by the trustor’s trust placed in them, and the freedom to carry out, or not carry out, the thing that they are being trusted to do. One must be ‘directly and favourably moved by the thought that A is counting on her’ (Jones 1996, p. 6).

AI may be programmed to have motivational states, but it does not have the capacity to consciously feel emotional dispositions, such as satisfaction or suffering, resulting from caring, which is an essential component of affective trust. An agent must be able to feel dispositions resulting from their actions; they must have mental states that are necessary for caring (Nahmias et al. 2020). If an agent cannot consciously feel anything, then it would be difficult to say anything matters to that agent, even if it can carry out actions similar to ours. AI may be able to act like us and have intelligence to carry out actions, while still not possessing the capability of being moved by those actions.

While we may be able to build AI to receive environmental input and stimuli, to detect appropriate responses, and program it to select an appropriate outcome, this does not mean that it is moved by the trust placed in it. While we may be able to program AI to replicate emotional reactions, it is simply a pre-defined and programmed response without possessing the capacity to feel anything towards the trustor. Artificial agents do not have emotions or psychological attitudes for their motives, but instead act on the criteria inputted within their design or the rules outlined during their development (Taddeo 2010, 2011).

Decisions made by AI do not matter to it. It does not have the ability to care about or be moved by the trust placed in it (Nahmias et al. 2020). While it may fulfil what the trustor is entrusting it with, it would certainly not because of any affective reaction towards the trustor. Without this, actions carried out by the AI cannot be grounded in trust, but are instead, acts based on reliance or predictability. In the affective account of trust, the trustee needs to freely act and be motivated by a sense of goodwill towards the trustor. Being moved by the trust being placed in one is an integral component of the affective account of trust, but narrow AI does not have this capacity, and thus cannot be something that has the capacity to be trusted.

##### Trust Based on a Normative Account

Normative accounts of trust emphasise what a trustee *should* do in a particular situation (Simpson 2012). When they breach this trust, they are violating the trustor’s expectations of what should occur and also their relationship with the trustor (Cogley 2012).

The trustee acknowledges the trust being placed in them and is aware of the potential betrayal of trust. They must use this information to determine if there are fit reasons to breach this trust, e.g. if the entrusted activity is immoral. For example, my friend asks me to keep his secret, but the secret is that he has a politician kidnapped in his basement. Telling the police would be a violation of his trust, and he may feel betrayed, but the breach of trust is justified. The agent should have the capacities to fulfil the entrusted activity, but they must also be free to choose if it is the morally right thing to do.^9^ The trustee needs to have the capacity to understand the relationship to the trustor, and the expectation that is being placed in them, to carry out the entrusted activity (Lord 2017, p. 23).

In the normative account of trust, the trustee also needs to be ‘an appropriate subject of blame’ during breaches of trust (Lord 2017, p. 23). The trustee needs to be able to understand and act on what is entrusted to them and be held *responsible* for those actions. Traditionally, artefacts have been used by full moral agents, so moral responsibility falls on those developing and using them (Himma 2009). However, what makes AI different is that they have a decision-making capacity, one that often surprises its creators (Gunkel 2012, p. 53). While AI is initially programmed to do certain activities, their actions often occur independently. Therefore, some claim that ‘the traditional ways of responsibility ascription are not compatible with our sense of justice and the moral framework of society because nobody has enough control over the machine’s actions to be able to assume responsibility for them’ (Matthias 2004, p. 177).

Matthias’ claim is that it would be unfair to hold the designers of AI responsible for damage or harm caused by them—because they can learn, act on their own, and developers do not have full control over them. However, even when there is a strong degree of independent decision-making, if AI causes harm, then someone needs to be held responsible for their actions (Goertzel 2002). Those who develop, create, and integrate AI into society should not be allowed to rescind their responsibility, simply because their creations act differently to how they were designed (Johnson 2006). This can be stated for any organisation creating a product for the market—that they have a social responsibility to ensure that their products do not cause harm to individuals within society and abide by the law. This is nothing new, and simply because AI has a greater level of autonomy than other artefacts, does not constitute an obfuscation of responsibility on those designing, deploying, and using them.

The main argument against assigning responsibility to organisations developing, deploying and using AI is that it will slow down progress because they will be more cautionary about what they design and release (Gunkel 2012). If making AI organisations responsible for harms caused by their technologies causes them to be more cautious and ensure that their technologies are safe to the end-user, then this is what should be done. This is already being applied in the field of self-driving vehicles. Manufacturers are acknowledging responsibility so that it does not slow down progress, but at the same time are implementing strong quality assurance procedures to reduce harm during testing (Ryan 2019c). So far, this has been a fairly effective procedure, despite some controversial incidents such as the Tesla Model S fatality in 2016 (Stilgoe 2018). If vehicle manufacturers could defer responsibility, when self-driving vehicles crash, onto the AI decision-making process, which would allow for speedier development and deployment, then they would probably do this. However, it would come at the peril of human safety.

If a vehicle at level 4 or 5 automation (NHTSA 2017) was the cause of a crash, because of a fault in its AI decision-making system, it would seem absurd for the vehicle manufacturer to shrug its metaphorical shoulders and say that because of the relative autonomy of the vehicle, they hold no responsibility for the accident. While Matthias’ position is philosophically-enticing for the AI industry, it is deeply problematic for policymakers, individuals, and society. Therefore, mechanisms should ‘be put in place to ensure responsibility and accountability for AI systems and their outcomes, both before and after their development, deployment and use’ (HLEG 2019, p. 19).

Referring to AI as trustworthy would inappropriately elevate AI, while disavowing the responsibility of those developing and implementing it: ‘Assigning responsibility to the artefact for actions we designed it to execute would be to deliberately disavow our responsibility for that design’ (Bryson 2018b, p. 21; see also Andras et al. 2018; and Bryson and Kime 2011). Companies should be held responsible for impacts of their AI and should instil measures to avert harmful impacts (HLEG 2019, p. 21). Responsibility should also lie with governments, industry leaders, research institutions (p. 33) and AI practitioners (p. 34).

Normative accounts of trust require moral agents to be held responsible for their actions, whether they carry out the activity they are trusted with or if they breach this trust. For AI to be classified as something that we can trust, it would require an explicit capacity to be morally responsible for its actions, in particular, the act that it is entrusted to carry out. AI does not have the capacity to be trusted according to the normative account of trust.

### Multi-agent Systems, Trust, and AI

It must be noted here that I am not excluding a trust directed towards individual human beings behind the development, deployment, and integration of AI, or the possibility of trusting the organisations developing, deploying and integrating AI. However, I am disputing the claim that the technology itself can be trusted or considered trustworthy. There are positions in the field that try to include AI as something that can be trusted in a very weak sense, often tying this trust to a trust in ‘multi-agent systems’, where AI is one of these agents.

Buechner and Tavani (2011), using Walker’s (2006) diffuse/default model of trust, claim that one can trust multi-agent systems that include humans, groups of humans, and also artificial agents—‘such as intelligent software agents and physical robots’ (Tavani 2015, p. 79). Walker stated that we trust particular zones and groups of people. She discusses larger groups or communities, such as cities, whereby people can follow practices appropriate for that place. There is a normative expectation on *people* to act in a certain way. This behaviour becomes habitual and ‘one simply engages in that behavior, with little or no conscious reflection’ (Buechner and Tavani 2011, p. 43).

Buechner and Tavani claim that this diffuse/default model of trust may be applied to AI, because it allows for distributing responsibility over a diverse network of human agents *and* artificial agents. As many acts of trust are grounded in non-interpersonal relationships, or mixed-relationships (i.e. interpersonal and non-interpersonal), then we should establish a type of trust that takes this into account. These mixed trust relationships, or multi-agent trust relationships, may take the form of trusting groups of individuals, organisations, and perhaps, AI technologies within that network of trust (Buechner and Tavani 2011).

Within the literature on the philosophy of trust, there is often disagreement over trust in organisations, institutions, and groups. Some argue that one can indeed place a trust in organisations as entities themselves, as they have a normative commitment towards us or we believe they are acting out of goodwill towards us. Others propose that these organisations are only a very complex form of interpersonal trust. When we refer to trusting an organisation, we are implicitly trusting the entire composition of individuals in that group to commit to the normative standards of their organisation. I will evaluate Buechner and Tavani’s position that we can trust AI in multi-agent systems, with these two positions in mind.

Firstly, Buechner and Tavani (2011) provide an example of an auction on eBay. There are many people engaging in this digital environment, such as the technical support staff, financial advisers, and developers. They contrast this to the type of trust placed in hospitals, department stores, or cities, where ‘the individuals with whom one has a trusting relationship need not be specified in advance, and need not ever engage in any kind of behavior that affects those who trust them’ (p. 43). However, this is still a zone of default trust in the organisation itself, and/or the other moral agents in these exchanges, regardless of their proximity or relationship to us.

It is a trust in eBay as a company to ensure that we are not scammed, and there are appropriate responses to those who do not respect their normative commitment to users. It is a trust in the individuals working in eBay who are designing the platform, who are running it, and who are ensuring that fair procedures are abided by. While users *rely* on the website to function correctly, the transaction process will work as it should, and eBay’s AI algorithms will show interesting related products; they can only *trust* the company, and/or individuals working within the company, for the same reasons discussed in the previous sections.

Regardless of the level of autonomy of the decision-making process of AI at eBay, its user-base places a trust in the company, and individuals working in the company, to ensure that they meet their normative commitments or are acting in the best interests of trustors when they trust them. Despite the complexity and multidimensionality of decision-making, it does not infer that one can, or does, trust the technologies that the company is using, no matter how advanced, autonomous, or intertwined they are within the business practices of the company.

While multi-agent relationships are a more complex combination of trust (interpersonal and institutional) and reliance (with the AI and other technologies being used), one should not attempt to conflate the two. It is also important to not allow AI to sneak into definitions of trust, simply because of the complexity and myriad of relationships taking place in multi-agent systems. Even if AI is making decisions and those decisions lead to a harm against the individual, the AI cannot be said to have breached the trust of the user, but rather, the developers or users breached this trust because they did not implement adequate procedures to prevent this from happening. This point is demonstrated in Walker’s airplane example, which Buechner and Tavani also refer to.

In this example, one receives poor service on an airplane. Buechner and Tavani (2011) propose that one may feel resentment towards the airline, but not specific individual agents (either human or artificial, one would assume) working for the airline, because one expects a normative commitment from the airline itself to provide a specific type of service. There is a normative expectation about what type of service they should receive on a flight. Therefore, ‘it would be foolish to say to someone that they should not resent the bad service of an airline, because you cannot resent the airline, but only the individuals who work for the airline’ (Buechner and Tavani 2011, p. 44). It is impossible to see why we would include AI as being something that we can trust, and not the organisation behind it, in a similar way to how we trust the airline and not the individual airline staff members working for it. Their own example appears to contradict their position that AI is something we can trust because of the myriad of networks that it may work within.

Overall, there is no reason to state that AI has the capacity to be trusted, simply because it is being used or is making decisions within a multi-agent system. If one is evaluating the trust placed in a multi-agent system as a complex interweave of interpersonal trusting relationships of those making decisions within multi-agent systems, one cannot trust AI for the reasons outlined earlier in this paper. If one is evaluating the trust placed in multi-agent systems as a trust in organisations, which AI is one component thereof, it has been shown, through the airline example, that this type of trust is not possible. These types of trust are directed towards the collective whole, rather than its individual parts, whether or not they are human or AI.

## Conclusion

Trusting relationships are those between trusted parties, whereas AI is a systematic group of techniques that enable machines to fulfil particular computing tasks: ‘AI is not a thing to be trusted. It is a set of software development techniques by which we should be increasing the trustworthiness of our institutions and ourselves’ (Bryson 2018a). Therefore, one needs to either change ‘trustworthy AI’ to ‘reliable AI’ or remove it altogether. The rational account of *reliability* does not require AI to have emotion towards the trustor (affective account) or be responsible for its actions (normative account).

One can rely on another based on dependable habits, but placing a trust in someone requires they act out of goodwill towards the trustor. This is the main reason why human-made objects, such as AI, can be reliable, but not trustworthy, according to the affective account.

In the normative account, the trustee must be held responsible for its actions, which AI cannot. Whereas, reliable AI places the burden of responsibility on those developing, deploying, and using these technologies.

Overall, proponents of AI ethics should abandon the ‘trustworthy AI’ paradigm as it is too fraught with problems, replacing it with the reliable AI approach, instead. The field should instead place a greater emphasis on ensuring that organisations using AI, and individuals within those organisations, are trustworthy.

## Footnotes

1 The HLEG consists of 52 industry, civil society and academic experts and was set up by the European Commission to establish ethics guidelines for the development, deployment and use of AI within the European Union. In their 39-page “Ethics Guidelines for Trustworthy AI” document, they use the terms “trust”, “trustworthy”, “trustworthiness”, “trusting”, and “trusted”, a total of 161 times.

2 By the socio-technical system they mean: ‘Analogous to questions of (loss of) trust in aviation, nuclear power or food safety, it is not simply components of the AI system but the system in its overall context that may or may not engender trust’ (HLEG 2019, p. 5).

3 There are some instances where it is claimed that there is no confidence in the trustee to fulfil the entrusted activity. However, these are much more the exception, rather than the norm. For example, in ‘therapeutic trust’, one may entrust someone with something, but do not believe that they will do it. They are hoping that this demonstration of their trust, and the practice of this relationship by the trustee, will cultivate their future ability to become trustworthy (Horsburgh 1960). For example, a parent may entrust their teenager with their house, while believing that their trust will probably be broken, but with the aspiration that they will become more trustworthy with time (McGeer 2008).

4 However, it must be made clear that having a confidence in the trustee to do X is only one component of the affective and normative accounts of trust, and is what differentiates them from the rational account of trust, and thus, what differentiates trust from reliability. This will become clear later in the paper.

5 Questions asked were: ‘By 2030, do you think it is most likely that advancing AI and related technology systems will enhance human capacities and empower them? That is, most of the time, will most people be better off than they are today? Or is it most likely that advancing AI and related technology systems will lessen human autonomy and agency to such an extent that most people will not be better off than the way things are today?’ (Anderson and Rainie 2018).

6 I may be vulnerable to the ridicule and shame of my secret being divulged; I may be vulnerable because of the supplies my friend is carrying during the hike or my safety if I have to help him along, or worse, he dies along the path; or I may be emotionally vulnerable to the pain and hurt my partner’s infidelity would cause.

7 For example, an individual who uses their non-autonomous car in a future dominated by self-driving vehicles is still vulnerable to the effects of the AI in these vehicles, despite not delegating any tasks to them (Ryan 2019c).

8 Conversely, I may place a greater level of trust in her the second time, leading to a greater sense of betrayal.

9 Therefore, the normative account of trust also explicitly requires the capacity of free will of the trustee. Thus, the arguments against the free will of AI in the previous section are also applicable here.
